# Editorial: Neuro-Immune Interactions in Inflammation and Autoimmunity

**DOI:** 10.3389/fimmu.2018.00772

**Published:** 2018-04-16

**Authors:** Niccolò Terrando, Valentin A. Pavlov

**Affiliations:** ^1^Center for Translational Pain Medicine, Department of Anesthesiology, Duke University Medical Center, Durham, NC, United States; ^2^Center for Biomedical Science and Bioelectronic Medicine, The Feinstein Institute for Medical Research, Northwell Health, Manhasset, NY, United States

**Keywords:** neuro-immune interactions, immunity, inflammation, autoimmunity, cytokines, antibodies

**Editorial on the Research Topic**

**Neuro-Immune Interactions in Inflammation and Autoimmunity**

The nervous system plays an important role in the regulation of immunity and inflammation (1, 2). However, neuronal integrity and brain function may be severely altered in inflammatory and autoimmune conditions (3–5). Recent studies have characterized neural pathways communicating peripheral inflammatory signals to the central nervous system (CNS), and brain- and spinal cord-derived circuitries controlling various innate and adaptive immune responses and inflammation (1, 2). A prototypical neural reflex circuit that regulates immunity and inflammation is the vagus nerve-based “inflammatory reflex” (6). Pharmacological and bioelectronic modulation of neural cholinergic circuitry in the inflammatory reflex has been successfully explored in preclinical settings of sepsis, arthritis, inflammatory bowel disease, obesity-driven disorders, diabetes, and other diseases (1). These studies paved the way to successful clinical trials in inflammatory bowel disease, rheumatoid arthritis, and metabolic syndrome (7–9).

Dysregulated release of cytokines and other inflammatory molecules may have a severe impact on brain function (10). Brain inflammation (neuroinflammation), imbalances in brain neuronal integrity, neurotransmitter release, and cognitive impairment are characteristic features of perioperative neurological disorders, sepsis, pain, liver diseases, diabetes, and other conditions characterized by immune and metabolic dysregulation (3, 11, 12). These findings and the characterization of brain-reactive antibodies and neuroprotective cytokines indicated new therapeutic approaches for treating inflammatory and autoimmune disorders and their neurological complications (4, 13, 14).

The collection of 19 papers on this research topic substantially contributes to improved understanding of neuro-immune communication and its therapeutic relevance. Yuki et al. elaborate on neuro-immune interactions within the gateway reflexes that regulate the entry of pathogenic CD4^+^ T lymphocytes in the CNS and neuroinflammation. They also point to the need of further characterization of the functional anatomy of these reflexes that will be of interest for their therapeutic exploration to alleviate local neuroinflammation in pathological conditions, including multiple sclerosis. Bonaz et al. review the role of the vagus nerve as a regulator of inflammatory processes in the gastrointestinal tract. They also elaborate on accumulating experimental evidence indicating possibilities to use electrical vagus nerve stimulation for therapeutic benefit in inflammatory bowel disease and other gastrointestinal disorders in preclinical and clinical settings. Innoe et al. outline new findings related to the neural vagus nerve control of inflammation with a specific focus on the kidney disease. They also summarize evidence that this neural regulation can be activated by the use of ultrasound and other modalities to alleviate inflammation and acute kidney injury in murine models. The splenic noradrenergic innervations are implicated in neuroimmunomodulation and are an important component of the inflammatory reflex through their functional cooperation with the vagus nerve. Hoover et al. provide insight into the anatomy of noradrenergic neurons in relation to leukocytes in the human spleen and experimental evidence for a significant splenic noradrenergic neuronal loss in patients who died from sepsis. These findings are of interest for further studies on the neural regulation of inflammatory processes in the spleen in the context of sepsis and other conditions. Fujii et al. present a thorough review of the cholinergic system, including choline acetyltransferase, acetylcholinesterase, acetylcholine, and muscarinic and nicotinic acetylcholine receptors in immune cells, such as macrophages, dendritic cells, and T and B lymphocytes. They also summarize findings about the functional role of this non-neuronal cholinergic system in the regulation of innate and adaptive immune responses. Valdés-Ferrer et al. evaluated the role of pyridostigmine, an acetyl-cholinesterase inhibitor, in a 16-week proof-of-concept open-label trial in HIV-infected patients with discordant immune responses. By using this strategy, the authors harnessed the cholinergic anti-inflammatory pathway and demonstrated that pyridostigmine can promote recovery of CD4^+^ T-cell counts by reducing T cell overactivation. Bosmans et al. discuss the role of cholinergic signaling, mediated through nicotinic and muscarinic receptors in the regulation of allergic inflammation, including barrier function, innate and adaptive immune responses, and effector cells responses. They also point to possibilities of exploring cholinergic regulation of type 2 immune responses in the treatment of allergic diseases.

Mader et al. outline how antibodies associated with autoimmune diseases lead to brain pathology and irreversible damage, specifically focusing on conditions such as systemic lupus erythematosus and neuromyelitis optica. They summarize some of the key mechanisms involved in these antibody-mediated pathologies and discuss the urgency to develop more appropriate and less invasive treatments for antibody-mediated diseases. Foster et al. outline the latest insights into the molecular mechanisms of the relationship between sensory neurons and immune cells, which can be protective, but also maladaptive. They elaborate on the role of nociceptors (noxious stimulus detecting sensory neurons) in the regulation of adaptive immunity with a specific focus on the involvement of nociceptors in the regulation of type 2 adaptive immune responses in airway inflammation. Gunasekaran et al. report on their discovery that peripheral sensory neurons of the dorsal root ganglia (DRG) of immunized mice contain antigen-specific antibodies. The authors use a combination of molecular genetic analyses, transgenic mice, and adoptive transfer experiments, to reveal that DRGs do not synthesize antigen-specific antibodies, but sequester primarily IgG1 subtype antibodies released by antibody-secreting plasma cells. In the broader context of the role of DRG sensory neurons in regulating antigen trafficking during immunization, these findings suggest that the nervous system may cooperate with the immune system to regulate antigen-mediated responses. Maurer and Williams describe the role of cholinergic signaling in cognition from the perspective of cholinergic modulation of microglia and astrocyte function, and the role of this regulation in Alzheimer’s disease and Parkinson’s disease. The colleagues focus on the cholinergic modulation of hippocampus-related neuroplasticity and memory processes and the involvement of the α7 nicotinic acetylcholine receptor in neuro-immune regulation in this context. Zaghloul et al. present new data about forebrain cholinergic dysfunction in parallel with neuroinflammation and peripheral immune and metabolic dysregulation in mice surviving cecal ligation and puncture-induced sepsis. These findings suggest that brain cholinergic signaling can be further explored as a therapeutic target in chronic sepsis illness, including cognitive impairment, functional disabilities, and alarmingly high mortality. Feng et al. demonstrate that preoperative exercise prevents the cognitive decline and neuroinflammation and increases the diversity of the gut microbiome in a model of tibia fracture with internal fixation surgery in rats with metabolic syndrome. These findings are of interest for considering exercising, acting as a microbiome modulator, in alleviating persistent cognitive impairment and neuroinflammation in postoperative conditions in high risk settings. Yang et al. used a laparotomy model in mice to report on the role of the blood–brain barrier in postoperative cognitive dysfunction. They found that anesthesia/surgery triggers endothelial dysfunction in an interleukin-6-dependent and age-associated manner, with significant cognitive impairments evident in 18-, but not 9-month-old mice. These data offer additional insights into the pathogenesis of postoperative complications like delirium and postoperative cognitive dysfunction. Berger et al. report results from a clinical study designed to evaluate neuroinflammation in patients with intracranial surgery receiving anesthetic maintenance with propofol or isoflurane. They found significant increases in cerebrospinal fluid (CSF) cytokine levels 24 h after surgery (as compared with levels before surgery), in parallel with increased CSF levels of tau, a neural injury biomarker, but no significant differences in terms of the anesthetic used. These results suggest that postoperative neuroinflammation in two anesthesia regimens may have a role in neural injury following intracranial surgery. Zhang et al. describe the neuroprotective effects of an annexin A1-derived small peptide on postoperative inflammation and cognitive function after cardiac surgery in rats. They report significant attenuation of microglial activation, cell death markers, and NF-κB activation after cardiopulmonary bypass with deep hypothermic circulatory arrest and, *in vitro*, using oxygen-glucose deprivation. These results provide evidence for the use of novel resolution agonists to treat inflammatory conditions in the perioperative space. Whittington et al. review the role of inflammation and specialized pro-resolving lipid mediators in neurological conditions and Alzheimer’s disease. Resolution of inflammation is well appreciated as an active process regulated by specialized pro-resolving lipid mediators derived from omega-3 fatty acids. The authors discuss on both preclinical and clinical evidence supporting the hypothesis that Alzheimer’s disease is a neurodegenerative disorder where failed resolution contributes to the disease process. Huh et al. outline the role of bone marrow stem cells or bone marrow stromal cells (BMSCs) in modulating chronic pain by acting in a paracrine fashion on peripheral and CNS sites to regulate inflammation. The authors review the mechanisms of substantial anti-nociceptive efficacy of BMSCs in preclinical and clinical settings and suggest possibilities for their use in the management of chronic pain. Pittaluga reviews the brain functional cross-talk between CCL5 (RANTES), released from glial cells and glutamate, the main excitatory neurotransmitter in the brain, in physiological and pathological conditions. She focuses on preclinical data demonstrating the role of CCL5 as a modulator of glutamatergic transmission in health and in demyelinating diseases with relevance to the onset of psychiatric symptoms and the progression of inflammation and demyelination.

Overall, the content of this research topic reflects the significant growth and advances in the field of neuro-immune interactions. It contributes to improved mechanistic understanding, which is interrelated with evaluating both pharmacological and bioelectronic approaches to modulate neural circuitry for therapeutic benefit preclinically and in human clinical trials. Further characterization of neural circuits and signaling mechanisms will be instrumental to inform the development of safer and more efficient therapeutic approaches for diseases characterized by dysregulated immune responses, autoimmunity, and inflammation.

## Author Contributions

Both authors wrote and approved the manuscript.

## Conflict of Interest Statement

The authors declare that the research was conducted in the absence of any commercial or financial relationships that could be construed as a potential conflict of interest.
